# Antibacterial resistance to antibiotics

**DOI:** 10.1017/S0950268824000530

**Published:** 2024-05-07

**Authors:** Norman Noah

**Affiliations:** Infectious Disease Epidemiology, Department of Epidemiology and Population Health, London School of Hygiene and Tropical Medicine, London, UK

The overuse and misuse of antibiotics have a long and shameful history. This dates back to the early 1950s when chloramphenicol came into use. It was the first broad-spectrum antibiotic, and even better, could be given orally. As a result, it was handed out like sweets for sore throats, minor cuts and almost anything with a fever until it was discovered to cause, in some, bone marrow suppression and death, as well as other side effects. Antibiotic overuse in more recent times in man and animal has led to a worldwide epidemic of bacterial resistance to antibiotics, and a never-ending cyclic race to keep discovering new ones, before they too persuade bacteria to become resistant to them. Bacteria too, seem to want to survive and do this effectively.

Dame Sally Davies has been a tireless advocate for the more controlled use of antibiotics. During her period as the Chief Medical Officer for England and now UK Special Envoy on Antimicrobial Resistance UK, she has been calling for evidence to develop the policies and actions we need across the globe. We at Epidemiology and Infection hope that this collection of papers, published recently, will help in some way towards achieving her goal during this important year when three high-level meetings will be held across the world.

I am most grateful to her, as well as to the two Associate Editors, Drs Ty Pitt and Pikka Jokelainen, for putting these papers, and many others, through the peer-review system and for their support towards enabling this issue to come to fruition.

